# Extracts and Gleanings

**Published:** 1876-06

**Authors:** 


### Extracts and Gleanings.

*On Pneumonia in Children.*—Dr. Wm. Stephenson says, in the *Edinburgh Medical Journal*, the prognosis of pneumonia depends much less upon the height of the temperature alone than upon the relation of pulse and temperature. It is, therefore, to this point our attention should be addressed, and to the other means whereby what I have termed tension may be estimated.

The use or warm compresses to the chest is of great value in all cases. Where tension is great, and where head symptoms are present, the whole body may be placed in a warm "pack." No means is more conducive to the comfort of the patient; the general uneasiness and sense of fullness of the system is allayed, and natural sleep induced. At the same time it must be remarked, I have never observed any decided change in the temperature or pulse from the use of the pack, but nevertheless the quiet and comfort afforded thereby is very evident.

Where further means are necessary to reduce tension, we have the selection of three valuable remedies—aconite, antimony, or ipecacuanha; but the good to be got from these is only within the first thirty-six or forty-eight hours. They should not be continued longer, and never, for any theoretical action upon the lung lesion, should they be given when the pulse is relatively high for the temperature. The large majority of cases, indeed, may be best treated by ipecacuanha or aconite, and acetate of ammonia. The value of aconite in acute inflammatory disease may be denied, if we judge of it by its power of lowering pulse and temperature, or cutting short the disease. Looking for its action in these quarters, I long disbelieved in its good effects, but have lately had my confidence in it renewed by observing the increased comfort of the patient, and the lowering of the tension where high, under its use. I have not yet, however, been able to record a definite action in the clinical chart, to change mere opinion into conviction. In case 5 (John Gill), where aconite was given throughout the febrile attack, it is worthy of note how the convalescence was prolonged by recurring high temperatures. Without assigning this to the effect of the unnecessarily prolonged use of the remedy, the circumstance must be noted at present in connection with it.

In a few rare cases, in healthy children, where the onset of the disease is associated with considerable dyspnoea, blood letting by leeches between the scapulae may be employed with undoubted effect; also, when pain in the chest is very severe, and preventing rest, a single leech to the seat of pain will afford great relief.

Where the pulse is high in relation to the temperature, quite an opposiie line of treatment must be followed. Here quinine, iron, and digitalis, singly or in varying combination, are the remedies indicated. In pneumonia, occurring amidst the sequelae of scarlet fever, I have seen good results also from belladonna. The selection must turn upon the due recognition of the constitutional state giving rise to the inordinate pulse. Various conditions may so act—the effect of the high temperature upon the nervous system, the amount of blood deterioration, a general cachectic state, or simply atony of the museular vascular system.

Several remedies may likewise be employed to minister to the comfort of the patient. I would especially mention morphia and spirit of chloroform.

Salines may be given from the first, in combination with the other medicines enumerated, and their use may be continued with benefit after the febrile stage has passed off, during the process of resolution. In this stage alone, and when the health of the child has been previously good, is any benefit ever to be derived from the use of mercurials. Where the process of resolution is tardy, or has been arrested, I have seen decided benefit from *small* doses of mercury.—*Medical and Surgical Reporter.*

*Scrofulous Sores.*—A Scotch clergyman states in the *Edinburg Medical Journal,* for March, 1876, that three grains of corrosive sublimate, in a pint of whiskey, constitute an infallible remedy in scrofulous sores or runnings. A rag dipped in this twice or thrice a day should be kept on the ulcers until healed.

*Treatment of Fractures by the Bran-Box.*—By Dr. A. Bates, M. D., of Viroqua, Iowa.—Allow me to call the attention of your readers to a mode of treatment for fractures, and other severe injuries of the leg and ankle, which I have succesfully used, both in simple and compound fractures.

The plan was suggested to me by the bran-box of Dr. J. R. Barton, as I had seen it in use in the Pennsylvania Hospital, thirty years ago. It was used then in cases ot suppurating compound fractures, to afford a comfortable bed for the limb, to absorb the discharges, and also, by covering the wound, to keep away flies.

The idea occurred to me that, by packing the bran firmly around the whole limb, any case of fracture could be treated, without any other dressing.

But I found, on trying it, that the bran was too elastic, and could not be made to secure the parts against motion. I, therefore, tried unsifted corn meal, which I found to answer in every respect. Since all are not familiar with Barton's bran box, I will describe my own method:

A piece of inch board, long enough to reach from the upper part of the thigh to a little beyond the foot, about seven inches wide at the upper end, and five inches at the lower end, will serve as a bottom of the box. A foot-board of the same thickness is nailed to the lower end, and two side pieces of thinner material, and at least seven inches wide, are firmly nailed to the bottom of the foot-boards, and the box is complete.

In applying it, a folded cloth should be laid in the upper end, and the bottom covered with meal an inch deep. Then, the surgeon holding the limb by the knee and ankle, an assistant places the box beneath, and the limb is let down into it, so as to rest lightly on the meal, and held firmly while meal is poured in and compacted under and around it with the hands, assisted by a blunt wooden spatula, until the parts are immovable, soft clothes being placed between the thigh and the upper part of the box. Care must be taken that the meal is well compacted around the knee and ankle; indeed, the whole limb must be equally secured.

To prevent disturbance from involuntary movements of the patient, a small pad may be placed on the knee, and secured by a bandage passed around the outside of the box. It will be found that a considerable amount of the surface may be left uncovered, and remedies may be applied to the injured parts if it is deemed necessary.

I have thought best to take the limb out of the box, and wash it thoroughly, at least once a week, but perhaps all that is really necessary is to remove that part of the meal in contact with the skin, as often as it becomes saturated with perspiration, and this can be done, a part at a time, without moving the limb.

Some of the advantages claimed for the above method are:

1. The limb can be examined at any time, without handling it.

2. The parts are immovable, and entirely at rest.

3. The circulation is not interfered with, since the pressure is not great, and is entirely even.

4. The dressing is more comfortable to the patient than any other that requires confinement to bed.

5. The material is cheap, and can be obtained almost anywhere.

6. The treatment is simple, and can be well applied by almost any one.

Various modifications might be suggested to meet particular cases, but these will suggest themselves to the surgeon, when occasion calls for them.—*Medical and Surgical Reporter.*

*Belladonna in Membranous Croup.*—By S. F. Gilbert, M. D., of Northumberland Co., Pa.

Having used the belladonna for some time, and in quite a number of cases with very gratifying results, and not having seen any recommendation of the remedy in this disease, it may be useful to put upon record a brief notice of some of my cases.

I first employed it hoping that it might prevent the further exudation of false membrane from its drying effect upon the mucous surface of the fauces and respiratory tract, and which has thus far been confirmed.

Case 1. J. S. æt. 18 months, was irritable and feverish on December 12th, with croupy cough and dyspnoea during night, which increased so that by the evening of 13th it was very severe, at which time I was called to see him. He was in great distress, his breathing very difficult, temperature high, pulse 160, croupous exudation on fauces. Gave an emetic of alum; when emesis had occurred, gave fld. ext. belladonna gtt. j; tr. aconite, gtt. j; tr. digitalis, gtt. v; spt. etheris nit. gtt. x, which was followed by sleep in about 20 minutes, with less difficulty in breathing. Then the following preparation was left: fld. ext. belladonnæ, gtt. iv; tr. aconite, gtt. viij; tr. digitalis, gtt. xl; spt. ether, nit. gtt. lxxx, with seven teaspoonfuls of water, one teaspoonful to be given every four hours; intermediate, tr. ferri chlor. gtt. v, were given in water; also left a reserve of fld. ext. belladonnæ to be given if difficulty of breathing increased, which was, however, not needed.

When I next saw him, 24 hours after, all the symptoms had abated, and the boy was apparently convalescent; treatment continued with longer intervals between doses: recovery from this on rapid.

Case 2. Clarie H., æt. 16 months, attacked Dec. 17th; treatment similar; recovery slower; general health more delicate

Case 3. Lydia S., æt. 2 years, attacked Dec. 27th; treatment similar, only no emetic was given at first. In this case recovery was rapid, but three days later was followed by an attack of acute bronchitis. The three days referred to were after I had discontinued my attendance for croup.

Case 4. Master H., æt. 14 months. This boy expelled a large piece of false membrane from trachea, so large that it almost strangled him, after which balladonnæ was given; other treatment similar as in preceding cases; no emetic was given; convalescence was more protracted. To be of benefit, the belladonna must be given in doses sufficient to produce dryness of the throat, and this effect maintained until the disease yields, at the same time great care is necessary to prevent the toxic effects of the remedy.—*Medical News*.

*Hæmatoxylum in Alkalinity of the Urine.*—Last year Mr. Cotton published in the *Lyon Medicale*, a communication relative to the antiseptic qualities of this substance. According to him, fermentation cannot take place in an infusion of the wood; and if a few chips are placed in decomposing urine its odour is removed. With the object of ascertaining whether this action could take place in the animal economy, M. Cotton gave to a man, aged 45, who had suffered for twenty-three years from distressing frequency of micturition, his urine being also very alkaline, from ammonico-magnesian phosphate, and albuminous, an infusion of half a drachm of logwood night and morning. In ten days the urine had become acid and free from crystals of the triple phosphates. The proportion of albumen was not changed, but there was a marked diminution in the frequency of micturition.—*Irish Hospital Gazette*, June 15, 1875.

*Local Application of Chloral Hydrate in Pruritus Vulvæ—Vicarious Menstruation by Sanguinolent Salivation.*—In *La Tribune Medicale* for December 19th 1875, a very interesting case of pruritus of the vulva is reported, which was treated by the local application of chloral hydrate. The patient, a young lady, whose age is not stated, had suffered for years from violent attacks of hysteria. Her menstrual flow had ceased, but each time when it should have appeared, she suffered from a sanguinolent salivation. On inquiry, Dr. Gelle, who reports the case, learned that she had suffered for some time from pruritus of the vulva, which was so severe as to necessitate her rising several times during the night to bathe in cool water. Dr. G., believing that the suppression of the menses (in the normal manner, at least) was due to the cold bathing, to which the young lady was obliged to resort, addressed his treatment directly to the pruritus of the vulva. He had recourse to chloral; a solution of ten grammes of chloral hydrate in one thousand grammes of water being employed as a wash several times a day Besides this, a tampon of wadding soaked in the mixture was placed between the labia. Under the influence of this treatment, so simple and so strictly local, the itching immediately became less, and gradually became so slight as to permit sound and continued rest. In the course of five days, the pruritus had entirely disappeared.

Dr. Gelle states that Dr. Laborde had previously obtained excellent results from the employment of this remedy in similar cases, and recommends its use to the profession.—*Va. Med: Monthly*.

It is pleasant to see that somewhere in the world, now and then, the physician's skill is property estimated. It may be remembered that Professor Depaul, of Paris, a few months since, was called to Brazil, to attend the Emperor's daughter in confinement, for which he received fifty thousand dollars. The reason for this extraordinary summons, as related by the Paris *Fiagro*, and copied in the *Medical Times and Gazette*, was, that after nine years of sterile married life, the princess, by following the treatment recommended by Depaul, became pregnant; but the child, unfortunately, was still-born. She became pregnant again, and this time, the Emperor solicited Depaul to come out himself and conduct the delivery, and he at last was persuaded to go. A baby weighing twelve pounds was, after thirteen hours labor, delivered with the forceps. It was an hour before it gave signs of life, and only after artificial respiration and the various methods of resuscitation had been tried. It is now a healthy child. The profession of Rio Janeiro received Professor Depaul quite coldly, and would render him no assistance. A great revulsion of feeling ensued, however, after the fortunate event had transpired, and Professor Depaul says, "My room was never empty from morning till night, and I was obliged, in spite of my determination to the contrary, to give consultations. In less than eight days fifteen thousand francs' worth of piaster were laid on my table as fees."—*Louisville Medical News*.

*To Cure Nail-biting*.—Children often get into the bad habit of biting their nails, and it is not wholly unknown among those of larger growth. A correspondent of the *British Medical Journal* having asked for a cure for this habit, a reply was given, suggesting the application of styptic colloid (tannin in collodion) in a thin line at the end of each nail. It dries quickly, forming a film, the bitter taste of which acts as a reminder. It must be reapplied whenever the hands have been washed.—*Boston Journal of Chemistry*.

*Sodic Sulpho-carbolate*—Its internal administration in the treatment and prevention of infectious diseases.—Dr. Brakenridge (*London Medical Record*, October 15, 1875—*Medical Times and Gazette*,) to ascertain the value of sulpho-carbolate of soda, carried out two sets of clinical observations:

1. The salt was administered in doses of from twenty to thirty grains, every two hours, to patients suffering from scarlet fever. Fifty successive patients were treated, all recovering. Nineteen were males, and the rest females. Average age was 17.5 years; average duration of the disease before treatment was commenced 4.4 days. In three cases only were there sequelæ, and in these the treatment was not begun until very late in the course of the disease. In contrast with these results, the author says that, of the twenty-four patients treated in the ordinary manner just preceding these cases for scarlet fever in the same wards, no fewer than six died.

2. Concerning the prophylactic virtue of the sulpho-carbolate, when given to healthy persons exposed to scarlet fever, diptheria and measles, the doctor says: It was given in seven families to twenty-two individuals exposed to the poison of scarlet fever; in three families exposed to the poison of diphtheria; in three families to eight persons exposed to the poison of measles. The dose varied according to age, from five to thirty grains, three or four times a day, or more frequently when well borne. In not a single instance did the disease extend beyond the individuals first affected.—*Detroit Review of Medicine.*

*Diphtheria.*—Dr. Hendrick's (*N. O. Medical Journal*) says For several years he has resorted to a plan of treatment suggested by the solubility of the false membrane in lime-water and lactic acid; uses the solution by inhalation. The usual tonic and supportive measures are kept up. Is better satisfied with this treatmen than any other. One case where there [illegible] complete aphonia recovered. Two similar cases died by suffocation, in his practice, before he had instituted this plan of treatment. Classes the muriated tincture of iron and chlorate of potash among the best tonic remedies.

*Atroplae Sulphas in Acute Myringitis.*—Dr. A. N. Ellis, Assistant Surgeon U. S. A. (*American Journal of the Medical Sciences*,) says: "Having been very familiar with the effects of the active principle of belladonna in painful and troublesome affections of the eye, I was led to use it in acute inflammation of the drum. About one year ago a soldier, standing near a cannon while the piece was being fired, suddenly experienced severe pain the head accompanied with hemorrhage from the ears. His sufferings were great. I saw him about six hours after the accident occurred. After carefully syringing the ear illumination showed fracture of the malleus and the seat of the hemorrhage. Aute inflammation of the drum supervened. Placing the patient in bed, a few drops of a solution of sulphate of atropia (four grains to one ounce of water) were dropped into the ears, six more upon the mastoid process. The effects were all h at could be desired. Since that time I have used the atropia in many cases of myringitis, and in every case with the best results. I am convinced that the prompt use of the remedy, conjoined with that of leeches and perfect rest in the recumbent position, will in almost every case give instant relief, thus arresting perforation of the drum and consequent suppuration."—*Canadian Journal of Medical Science.*

*Obesity Treated by Liquor Potassæ.*—Dr. Foot, having a case of extreme obesity, rendering the patient incapable of locomotion and threatening speedy death, gives the following record: Two suggestions presents themselves in order to prevent a fatal issue—first, to lessen, if possible, the present accumulation of fat; secondly, to restore muscular activity. The patient went out of the hospital one an l a-half pounds heavier than when he was admitted, and that was accounted for by the loss of fat being replaced by the regaining of muscle. Enforced muscular exercise was made part of his treatment, as soon as the patient was able to move about. The treatment consisted in the administration of two drachms liquor potassæ, three times a day in a teaspoon of milk, with drachm doses of fluid extract of fucus vesiculosus. He was restored to the use of his lims, so that he could walk long distances and perform light labor.

*The Longevity of Physicians.*—Editors *Medical aud Surgical Reporter:* We have been making a collection of facts concerning the ages of physicians, the result of which we give below. Commencing with the twelfth volume of the *Reporter*, and ending with the issue of May 2d inst., we have found 892 obituary notices of physicians, whose ages were given. We took *one* from the *Northwest Medical and Surgical Journal*, and *seven* from the report on Necrology made to the American Medical Association at its meeting in St. Louis, making in all 900. The average age of this number is 56.57 years; the minimum age being 21 years, and the maximum age 103 years.

The number of deaths between the ages of 20 and 30, 63, or 7 per cent; between the ages of 30 and 40, 125, or 13 8-9 per cent; between the ages of 40 and 50, 104, or 11 5-9 per ce it; between the ages of 50 and 60, 145, or 16 1-2 per cent; between the ages of 60 and 70, 187, or 20 7-9 per cent; between the ages of 70 and 80, 163, or 18 1-9 per cent; between the ages of 80 and 90, 96, or 10 6-9 per cent; between the ages of 90 and 100, 16, or 1 7-9 per cent; over 100, 1, or 1-9 per cent.

Respectfully, etc.,

H. WARDNER, M. D.,
H. J STALKER, M. D.

*New Use of Quinine.*—The unquestionable and remarkable influence of quinine over white corpuscles, when directly brought into contact with them, has been utilized in the topical treatment of supperations. Morlard, of St. Louis (*Pract.*, November, 1874,) injected 6 grains of quinine, dissolved in two-thirds oz. of water, into the cavity of a suppurating pleura. The discharges of pus rapidly diminished, as it had not done under carbolic acid treatment. An ulcer on the leg, of two years standing, and associated with initial heart disease, was treated with an ointment of quinine, 10 grains to the ounce. In two or three days, suppuration diminished, then healthy granulations appeared, and the ulcer was rapidly healed. The third experiment, equally successful, was on a mammary abscess, treated by an injection of ten grains quinine to f oz. j of water.—*New Orleans Medical and Surgical Journal.*

*Topical Application of Medicated Ice.*—Dr. Edward Martin, in a recent number of the *Lancet*, recommends the following method for overcoming the difficulties of the scarlatinal throat in young children. He says: "Where I could not persuade the child to submit to the sulphurous acid spray, a frozen solution of the acid, (or some other antiseptic) has been my chief resource. Though, of course, not so tasteless as pure ice, the flavor is much lessened by the low temperature, and probably also through the parched tongue very little appreciating any flavor whatever. The process of making it is very simple. A large test-tube immersed in a mixture of pounded ice and salt is the only apparatus required, and in this the solution is easily frozen. I have tried the three following formulæ, all of which answer, though I think I prefer the first:

1. R. Sulphurous acid........ ½ fl. drachm

Water....................7½ "

Mix and freeze

2. R. Chlorate of potassa...1 scruple.

Water....................1 fl. ounce.

Dissolve and freeze.

3. R. Solution of chlorinated soda...........½ fl. drachm.

Water .....................1 fl. ounce.

Mix and freeze.

"Boracic acid, salicylic acid, or any other harmless antiseptic, with not too much taste, would doubtless answer as well as those indicated.

*Sweating Feet.*—The following is recommended by Hager:

| | | |
|---|---|---|
| Burnt alum.... . ...................... | 5 | drachms. |
| Salicylic acid.................. .... | 2½ | " |
| Wheat starch.......................... | 15 | " |
| Powdered talc......................... | 50 | " |

Mix thoroughly. To be dusted on the feet and in the stockings. Various lotions are applied with success, such as weak solutions of tannin, acetate of lead, sulphate of zinc, etc.—*The Druggists' Circular and Chemical Gazette.*

*Remedy for Bowel Affections.*—Dr. M. J. Eley, of La Fayette, Ala., writing to *The Medical and Surgical Reporter*, recommends the following preparation as being a safe and efficient remedy. He says: "In the number of your journal for September 7th, 1872, Dr. Downing called attention to the following 'New combination for bowel affections,' asking other physicians to try it and report the result:

The formula is as follows:

R.—Socotrine aloes, powd.,
Sulph. potash, powd.,
Bicarb. soda,........... aa......oz. j
Cloves, powd.,...................oz. ss. M.

Divide into twelve powders.

"To one of these powders he added three tablespoonfuls of boiling water, giving the whole at one dose—as hot as could be borne—to an adult. To be repeated every hour. To children one teaspoonful to be given every half hour.

"The remedy was warmly recommended in all cases of cholera morbus, diarrhœa, and dysentery.

"Since I first noticed the above mentioned combination, now four years, I have been using it, with uniform satisfaction, in the above-named troubles as seen in children, especially in what we usually term 'summer complaints' and 'teething diarrhœa.' My plan of administering is somewhat different from Dr. Downing's; for instance, I add one teaspoonful of the powder to a teacup of boiling water; when cool, give a teaspoonful every hour till the discharges become normal in frequency and color." *The Druggists' Circular and Chemical Gazette.*

*Vanillin from the Pine.*—A very important chemical discovery has recently been made in the laboratory of Professor A. W. Hoffman, at Berlin. He has produced from the cambium juice of certain trees of the *Coniferae* order a crystalline substance, to which he has given the name of *vanillin.* This vanillin is a perfect substitute for vanilla, and is a very remarkable addition to the series of the popular and economic triumphs of modern chemistry.—*Medical and Surgical Reporter.*

*Diphtheria.*—Dr. Crawcour (*N. O. Medical Journal*) says The disease is undoubtedly constitutional, and the best remedia agents are those which act chemically on the zymotic cause. Must say, however, that he has heretofore been unsuccessful in the treatment of the disease; has recently, however, had a case to recover, where, owing to the gravity of the attack, he expected death: was called to see a child about one year of age; there was fever, no difficulty in swallowing; a leathery yellowish deposit on both tonsils; great depression. Ordered the throat to be mopped out with a solution of carbolic and salicylic acids, applied quinine innunctions to the body, and gave internally a strong solution of chlorine; gave iced milk freely. The child is now convalescent.

In other cases has used carbolic acid or salicylic acid alone, and has been unsuccessful. Has had two cases recover under the internal administration of bromine—one-fifth of a drop every hour. Advised, when administering bromine, that it be combined with small quantities of bromide or iodide of potassium, to insure its solubility. The solution should be kept in the dark, as light decomposes the bromine into hydrobromic acid.

*Soft Hands.*—To preserve the smoothness and softness of the hands, keep a small bottle of glycerine near the place where you habitually wash them, and whenever you have finished washing, and before wiping them, put one or two drops of the glycerine on the wet palm and rub the hands thoroughly with it as if it were soap, then dry lightly with a towel. Household work and bad weather will not prevent your skin from being smooth and soft if this plan of using glycerine is followed.—*The Druggists' Circular and Chemical Gazette.*

*Removal of Foreign Bodies from the Ear.*—Let the surgeon take six inches, or as much as he pleases—it is always handy and plenty of it—of horse hair, double it into a loop; then having the patient placed on his side, pass the loop into the ear as far as it will go; turn it gently, and at first or second withdrawal, the foreign body will come out in the loop. It gives no pain, and cannot do damage.—*The Clinic.*

*Apomorphia as an Emetic in Children.*—Apomorphia has been known for several years as one of the alkaloid principles contained in opium. It is devoid of narcotic properties, if carefully prepared, and acts principally as an emetic. Dr. W. F. Duncan, of Randall's Island Hospital for Children, (*Medical Record*, August 7, 1875,) extols it as the most efficient and prompt of all emetics, especially for children, and in cases where it is desirable to evacuate the stomach of poisons. It is particularly applicable for hypodermic use, producing emesis in this way in from two to four minutes. One-tenth of a grain is a full dose for an adult, and one-fiftieth of a grain for a child one year old. Dr. Duncan bases its merits on the following grounds: 1. Rapidity of action. 2. Absence of danger from an overdose. 3. Lightness of secondary effects. 4. Shortness of period of nausea. 5. Ease of administration. The preparation commonly employed is the hydro-chlorate, and the English preparation is preferred to the German, because the latter has been known to contain an impurity of morphia. For hypodermic use, it is advised to dissolve it in water, with a small quantity of glycerin and alcohol.—*Pacific Medical Journal.*

*Pruritus.*—For the intolerable pruritus common in fall and winter, many physicians use Dr. L. Duncan Bulkley's prescription, given in the *Transactions* of the American Medical Association, viz:

*Unguentum Anti-pruriticum.*

R. Pulv. gum camphor,<br>

Chloral hydrat, } Of each 1 drachm.

Grind well together in a mortar, till they form a fluid, and add slowly, simple cerate, one ounce.

Apply to the part affected, *but not to be used if the skin is at all broken.*—*Med. Brief.*

*Alcoholism treated by Strychnia.* — (*Apoth. Zeitung*) This remedy has long been employed by the laity, after excessive indulgence in spirituous liquors, and W. Morey has, for some time, administered it with excellent results in delirium tremens. He is convinced that strychnia is the best antidote for alcoholism. He gives either the extract or tincture of nux vomica.

*Strychnia vs. Opium.*—A very interesting case has just been reported to us, showing the antagonism of strychnia to opium. A gentleman, for suicidal purposes, took between 40 and 50 grains of gum opium. Three hours later, when Drs. Harris, Ham, and other physicians, were called, the man was apparently in a dying condition. His extremities were livid and pulseless, while his respirations were only eight per minute. As consciousness was completely gone, and the opiate probably mostly absorbed, the stomach pump and the usual remedies were inapplicable. At the suggestion of Dr. Harris, not approved nor yet positively condemned by the other physicians present, 40 minims of a saturated tincture of nux vomica were injected hypodermically, in quantities of about eight minims, at five different spots, over the chest, arms, etc. In fifteen minutes the patient sat up, and in twenty minutes, walked about the room; and did not again sleep from the effects of the opium. No symptoms of strychnia poisoning appeared. The tincture employed was prepared by Dr. Harris, who thinks it was fully as strong as a fluid extract should be.—*Chicago Medical Times.*

*Quinine Gargle in Sore Throat*—Dr. George Johnson gives the formula below as of excellent effect in sore throat, even severe diphtheritic cases:—

R—Quinia sulphatis.......... 18 grains.
Acidi sulphurici diluti.... 42 drops.
Aquæ, ad..................... 6 fluid ounces.

He adds, I have found the quinine solution useful as a wash, in aphthæ, stomatitis, and other affections of the mouth; but my experience of it in these cases has been limited by the difficulty attending its use in childhood, owing to its very bitter taste.—*Medical Brief.*

*When Not to Sound.*—Dr. Cohnstein gives the following contra-indications for the use of the uterine sound, in the order of their importance:—1. If there is a suspicion of pregnancy; 2. If there be great general irritability; 3. If the uterus be very sensitive; 4. In inflammatory condition of the uterus and its adnexa; 5. When the uterus is fixed by adhesions.—*The Medical and Surgical Reporter.*

*Subcutaneous Injection of Carbolic Acid in Rheumatism.*—Dr. Senator lately brought before the Berlin Medical Society the Local Employment of Subcutaneous Carbolic Acid Injections in Rheumatic Polyarthritis recommended by Kunze. At first he used a solution of one per cent., and later, of two or three per cent., injecting this in the vicinity of the various joints affected. These injections had never been attended by any ill consequences, and had often given rise to the disappearance of the pain and other local symptoms. Complications and relapses were, however, not prevented.

Dr. Goldbaum had, for several years, been employing subcutaneous injections in articular rheumatism, as also in muscular rheumatism, and the anæsthetic effect has been remarkable, continuing for five or six hours; not only is the pain relieved, but the joint becomes movable, such mobility remaining as long as the pain is absent. He believes that, in several cases, a favorable influence is exercised on the general course of the disease. The efficacy of the practice is especially seen in muscular rheumatism, so that patients who were motionless from lumbago were, after the injections, able to move; and after these had been repeated several times, the affection disappeared.

Dr. Waldenberg called attention to an old and very simple palliative that had very seldom failed him, viz.: pouring ether over the part. Applied as ether spray he had found it less efficacious.—*Nashville Nedical Journal.*

*Hair Restorative.*—Dr. Tilbury Fox gives the following formulæ for stimulating the scalp: 1. Glycerine, 3 drachms; lime-water liniment, 4 ounces; tinct. cantharides, 3 drachms, M. 2. Distilled vinegar, 3½ ounces; tinct. cantharides, 6 to 7 drachms; rose-water, 3½ ounces. M. 3. Strong ammonia liniment, ½ ounce; castor-oil, ½ ounce; purified spirits or turpentine, ½ ounce; white precipitate, 15 grains. M. Brush into the scalp with a hard nail-brush until irritation is set up. 4. Tinct. cantharides, 1 ounce; distilled vinegar, 1½ ounces; glycerine, 1½ ounces; spirits of rosemary, 1½ ounces; rose-water, 8 ounces. M. To be sponged well on the scalp night and morning.—*New Remedies.*

*Opium Treatment of Diabetes.*—The treatment of diabetes with opiates, introduced by McGregor, in 1837, has yielded different results in the hands of different physicians. A writer in the *Chicago Medical Journal* strongly asserts that opium, or morphine rather, exerts a direct influence upon the excretion of sugar, diminishing its quantity or making it disappear entirely. But large doses must be given, and to the administration too small doses does he attribute the ill success of other practitioners.

He bases these assertions on the clinical observations of fourteen cases, in which satisfactory results were obtained by the employment of large doses of morphine. "In most of the cases the disease had lasted a long while, and visibly influenced the nutrition of the patient. None of the patients were put on an absolute animal diet, but always a small amount of bread was allowed. In all cases, a decrease in the quantity of sugar in the urine ensued, sooner or later, upon the administration of morphine, and if the dose was gradually increased, the excretion of sugar ceased temporarily. At the same time the excess of urine decreased, the thirst subsided, and the nutrition of the patient improved. With most of the patients, it is true, this improvement lasted only as long as morphine was given; in one case, however, no sugar could be found in the urine, eighteen months after the treatment had been stopped. The doses of morphine were gradually increased to as much as three grains daily, until the urine did not contain any sugar; were then discontinued, to be given again when the sugar re-appeared, to the amount of two or three per cent., or moderate doses of morphine were continued, to keep the excretion of sugar below two per cent." The remedy is very well tolerated by such patients, and its narcotic influence does not appear until very late, but it causes an obstinate constipation, which, however, can be counteracted from time to time, by an enema or the use of rhubard, or aloe.

This writer does not feel prepared to assert that this treatment can cure every case of diabetes, but he thinks it may always lengthen the life of the patient.—*New Remedies.*

*Pencils of Nitrate of Zinc.*—Pencils and cones of nitrate of zinc, which have come into use of late for cauterizing purposes, may be made as follows: Dissolve good commercial zinc (Lehigh) in nitrate acid (sp. gr. 1.200,) until the latter is saturated; separate the solution from the undissolved zinc, and while still warm, add one part of precipitated carbonate of zinc for every thirty-two parts of zinc in solution. The carbonic acid of the carbonate of zinc is transferred to the iron, which contaminates the commercial zinc and is present as ferric nitrate, and an additional quantity of nitrate of zinc is formed; the heat, however, causes the decomposition of the ferric carbonate, the carbonic acid escapes, aud ferric oxide, together with the excess of carbonate of zinc is deposited. Filter the solution, which must be considerably diluted with water to prevent it tearing the filter, and evaporate it on a sand-bath, until it appears as a quiet, fused mass, but yet liquid. Should the evaporation have been conducted too far, which is indicated by the escape of yellowish fumes, the vessel should be removed from the fire, allowed to cool, and a quantity of very dilute nitric acid added, after which it is again to be evaporated to the proper point. This fused liquor, which must not be so hot as to ignite paper, on which a few drops have been allowed to fall, is poured into paper moulds about four inches in length, made by rolling paper around glass rods or lead pencils, pasting the edge and closing the bottom. No oil or fat of any kind must be used, as the paper will invariably take fire in this case. When the sticks are hard they are enclosed in glass tubes, and the latter well corked. For use, a small quantity of the paper is removed from one end by means of a knife, and, if desired, the end of the pencil may be pointed.—*New Remedies.*

*On Jaborandi.*—Dr. Felippo Cesari, of Rome, in the *Gazeta Medica*, of that city, has an article entitled "Physiologicel Experiments and Clinical Notes on the Polycarpus Pinnatus, or so-called Jaborandi," in which he comes to the following conclusions: 1. That the jaborandi of Continho has an elective action on the salivary glands. 2. That it has a secondary action, but less constant and copious, upon the sudoriparous glands. 3. That it has a well-marked diuretic action. 4. That it produces no effect whatever on the circulation, nor on the generation of heat, nor (except in an unimportant degree) on the respiration. 5. That its action is most efficacious when it is injected into the veins. 6. That its after effects are distinct debility, with an imperious thirst, directly proportioned to the action of the emunctories. These effects, however, are transitory, and soon disappear under restorative diet.—*Medical and Surgical Reporter.*

*Lime Juice and Pepsine.*—Dr. Archer Farr, of London, says the *Chemist and Druggist*, has started the idea of administering pepsine in cases of dyspepsia in conjunction with lime juice. He believes that the anti-scorbutic value of the latter is due to its power of dissolving certain portions of food; and having regard to the fact that the gastric fluid invariably contains acid of one kind or another, he concludes, not unreasonably, that the acid may perhaps claim some credit in promoting the digestive process. This combination, therefore, theoretically suggested, he has used practically, he says, with the best results; and his preparation, which seems to be a mixture of pepsine and lime juice, is now offered generally to the trade and to the public·*The Druggists' Circular and Chemical Gazette.*

*Chloral in Pityriasis.*—Dr. Martineau states, in a French cotemporary, as the result of a large experience, that chloral is a very efficacious, if not certain means of treatment, in this rebellious affection. The solution he used was of the strength of about forty grains to each ounce of water, and this he applied to the scalp each morning, by means of a spunge, using slight friction, and allowing it to dry. If the disease is recent, and the lotion is uninterruptedly used for a month, he predicts a certain cure. In the chronic and more obstinate cases, he recommends the continuance of the application of the solution, until the disease disappears, as its daily use produces no inconvenience, whilst it relieves the itching.—*Medical and Surgical Reporter.*

*Treatment of Albumenuria*.—In acute and chronic renal disease, the broad principle to be acted upon is, clearly, to relieve the kidneys as much as possible of their work, for a certain time, by exciting the skin, bowels, and lungs, to perform, vicariously, the renal functions. External warmth is the best diaphoretic, in this instance, especially the hot-air bath. The alterations in the blood giving rise to albumenous urine may be best reached by iron, which has a remarkable effect upon the re-absorption of serum. The undried sulphate is the best. To this should be added the phosphates, which are the true remedial agent in this condition.

The following formula we have found remarkably efficient:

R—Phosphate of soda.... .......................... oz. j.
Sulphate of iron (undried) .................. oz. j.
Iodide potassium .............................. oz. ss.
Water .............................................. fl oz. x.
Tablespoonful three times a day.

DR. T. O. SUMNER, JR.

*Pills for Obstinate Neuralgia.*—The *Bordeaux Medical* gives the following formula for obstinate neuralgia, especially ileo-lumber neuralgia:

R.—Valerianate of Amonia,
Quinine, aa gr. xxx.

Make into twenty pills and take from two to ten of them each day, increasing one pill per diem. After taking these pills for ten days, suspend their use for five days.—*Medical and Surgical Reporter.*

*A New Preparation of Ergot.*—A very active preparation of ergot, which is particularly adapted for subeutaneous injection, is suggested by Dr. Wernich, of Berlin, who proposes to exhaust the drug with ether, strong alcohol, and finally with water. The influsion is then dialized through parchment paper, and the solution evaporated. This extract, after acidulation with sulphuric acid, was mostly soluble in alcohol, and when again carefully neutralized by soda, yielded all its active properties to weak alcohol—*Apotheker Zeitung—American Journal of Pharmacy.*

*Quinetum.*—A preparation of the whole alkaloids, separated from East India red bark, has been used, for some time, in the Indian hospitals, as well as in private practice, with great success. As manufactured by Thomas Whiffin, an English manufacturing druggist, it is a fine, granular, non-adherent powder, of a pale buff color, which, dissolved in dilute sulphuric acid and water, is a clear brown liquid. Sulphate of quinetum is a white crystalline body, with a faint pink tinge, greatly resembling sulphate of quinia. It dissolves readily indilute acids, and the solution exhibits a strongly marked fluoresence. It is said that the price of quinetum is about half that of quinia. The same firm is manufacturing a citrate of iron and quinetum.—*New Remedies.*

*Formula for the Troublesome Cough of Phthisis.*—

| | | |
|---|---|---|
| R.—Potassii bromidi | } | Of each 1½ drachms |
| Potassæ chloratis. | } | |
| Ammoniæ muriatis | } | |
| Syrup tolutani | | 4 ounces. M. |

Tablespoonful every two or three hours.

| | |
|---|---|
| R.—Tincturæ opii champhoratæ | 1 ounce. |
| Tincturæ belladonnæ | 1 drachm. |
| Tincturæ hyoscyami | 2 drachms. |
| Spiritus Lavendulæ comp | 1 drachm. M. |

Ten drops on a lump of sugar every hour until cough is relieved.—*Charity Hospital, New York.*

*Gangrene; Treatment with Salicylic Acid.*—(*New York Medical Jonrnal,* January, 1876).—Dr. N. G. McMaster has used this acid as an application to gangrenous surfaces, with marked benefit in keeping down the intolerable odor. One case, particularly, was satisfactorily treated in this way. Bromine had first been applied, then carbolic acid, then poultices of charcoal, but the odor was nevertheless sufficient to exclude the patients from the ward. The salicylic acid in powder was then either dusted on the surface or bloom into the cavities, as necessity indicated. After the thorough use of this agent the offensive odor was completely controlled.—*Philadelphia Medical Times.*

*Pumpkin Seed in Treatment of Tape Worm.*—Dr. Jones writes: I took two ounces of decoction of pumpkin seed, rubbed them up with one ounce of white sugar, and added eight ounces of water, and four ounces of mint water, the whole making a delicious emulsion of fourteen ounces. I directed him to eat no supper, and to take the emulsion the next morning before breakfast, in three equal doses, at intervals of fifteen minutes, and to take the oil mixture (I had prepared him an ounce of castor oil in emulsion) two hours after the last dose of the emulsion. He took the last dose of the emulsion at 6 o'clock. I called at 10, found him asleep, and the oil mixture not taken. I gave it to him then, and called again at 12. He had had no action from his bowels. I repeated the oil mixture and added one ounce of croton (?) to it. I called again at 2, and still the bowels had not moved. I advised a lavement of soap and warm water, which was followed in ten minutes by a very copious action from the lower bowel, but there was no sign of the worm. The patient had taken neither supper, breakfast, nor dinner, and at my suggestion he took a plate of soup and three biscuits of unbolted flour. At 2½ o'clock, eight hours and a half after taking the last dose of emulsion, he had a free action through the alimentary tract, and here is a tænia solium, twenty-one feet long and entire, the result of the treatment.—*The Richmond and Louisville Medical Journal.*

*Silicate of Potash in Erysipelas.*—*La France Medicale* gives an interesting account of this method of treatment, devised and successfully carried out by Professor Alvarenga, of Lisbon. It is the result of physiological experiment, and not of mere empiricism; the Professor having tried the drug first on himself. When applied to the skin, immediately a sensation of coolness and retraction is felt, the skin becomes pale, most markedly so if it has previously been red and congested, and thermometric observations show a decrease in temperature. Forty-eight cases are reported treated by this method alone, all of which recovered in periods varying from four to six days from the commencement of the application.—*The Medical and Surgical Reporter.*

*Epistaxsis.*—Dr. Prince (*St. Louis Medical Journal*) says: I had some years ago a case which resisted all medicine and local application, the bleeding being only temporarily arrested by plugging. Tartar emetic produced no apparent effect, gallic acid was powerless and chloride of iron failed. Finally veratrum viride was given, and as soon as the pulse became retarded the bleeding ceased and did not return. In this case cold and pressure were unavailing.

Soon afterward a girl of about twelve years, had a tooth drawn, and the dentist had exhausted the expedient of pressure and cauterization, and the adjacent mucous membrane had begun to show hemorrhage specks. Veratrum was given, and as soon as the heart came under its control the bleeding ceased and did not return.—*Journal of Materia Medica.*

*Diphtheria.*—Dr. Dell 'Orto (*N. O. Medical Journal*) says: He pours in a glassful of water, from 20 to forty drops (according to the age of the patient,) of pure perchloride of iron. Of this mixture he makes the patient drink about two teaspoonfuls every ten, fifteen or twenty minutes, (according to the gravity of the case,) and he continues, day and night, during four or five days. Generally, a patient can drink from seven to ten glassfuls of this solution in the twenty-four hours, which makes an average of 140 and 360 drops of perchloride iron. The nourishment must be strictly reduced to pure cold milk, which ought to be given as regularly and often as the solution.

*Aphonia.*—Dr. Gemer gives a case of complete aphonia in a young lady from exposure to cold. Many remedies were tried in vain for three months, when she recovered her voice in three days from the inhalation of ammoniacal vapor, disengaged from a mixtue of a solution of muriate of ammonia and carbonate of potash. One would suppose that it would have been easier to inhale, or smell of Aqua, or Carbonate of Ammonia. I have cured many cases by the inhalation, and especially by the internal administration of Ammonia; and have often relied successfully on the Aromatic spirits as in the case of the celebrated singer Mario.—*West Virginia, Medical Student.*

*Blackbery Brandy or Cordial.*—

| | |
|---|---|
| Blackberries, crushed | 1 gallon. |
| Brandy | 1 " |
| White sugar | 2 pound. |

Macerate the berries with the brandy for five or six days, express the liquor, add the sugar, and, after two weeks, decant or filter. In winter, the blackberries can, probably, be replaced by the preserved juice in proportion, say, of about four pints of juice for one gallon of berries.—*The Druggists' Circular.*

*Medical Value of Eucalyptus.*—Dr. Paul Boyce says, in the Virginia *Medical Monthly*: A case of ague and fever, which had been treated with quinia, arsenic, etc., was cured in four days, by simply taking the essence of eucalyptus (prepared from the oil distilled from the leaves) drachms ij three times a day. I believe we possess in the eucalyptus, a remedy not inferior to the cinchona alkaloids. The oil applied to the nerve of a tooth soon destroys its sensitiveness and quiets the pain. In purulent catarrhal affections of the urethra, *it acts like a charm.*—*Medical and Surgical Reporter.*

*Improved Cathartic Pills.*—The subjoined formula has been recommended:

| | |
|---|---|
| Podophyllin, | |
| Compounded extract of colocynth, | |
| Extract of jalap, of each | 30 grains. |
| Extract of hyoscyamus | 10 " |
| Powdered capsicum | 6 " |
| Oil of peppermint | 6 drops. |

Mix. To make 60 pills. Dose, from one to three.—*The Druggists' Circular and Chemical Gazette.*

*Tincture of Poison Oak.*—

| | |
|---|---|
| Fresh leaves of poison oak | 4 ounces. |
| Alcohol | 3 fl. " |

Macerate for fourteen days, express and filter under cover. Dose, from three to ten drops in water. It is used in epilepsy, but the greatest care is recommended in its administration. As the active principle is volatile, the tincture must be kept in closely stopped vials.—*The Druggists' Circular and Chemical Gazette.*

*Diphtheria.*—Dr. Rhett, in *Charleston Medical Journal*, recommends the following formula as highly effacacious in diphtheria:

R. Chlorate potash, from.................... dr.ii to dr.iv
Hydrochloric acid, from .................. mxx to mxxx
Glycerine ........ ............................ f oz.ii to f oz.iv
Water............................ ..........foz.xii to oz.xiv

Keep in the dark, and give a tablespoonful to adult every two or three hours, and gargle with same between the doses.

*Antispasmodic Cough Mixture.*—

R. Chloral hydrat.........................................dr.iij.
Potass. bromid.........................................oz. ss
Tinct. opii. camp........................................oz.i.
Pulv. acaciæ..........................................dr.ij.
Syr. Pruni Virg........................................oz.iv.
Aquæ puræ.................................. ...oz.ii.ss.

*Syrup of Salicylic Acid.*—In giving this acid the annexed formula, for a syrup, has been suggested:

R. Salicylic acid.........................................dr.ss.
Oil of sweet almonds....................................dr.x.
Gum arabic.............................................dr.x.
Syrup of almonds......................................dr.xij.
Orange-flower water...................................dr.xij.

—*The Medical and Surgical Reporter.*

*Infantile Vomiting.*—Purgatives or ordinary astringents being either premised or contra-indicated, a valuable remedy is known in quarter or half drop doses of dilute hydrocyanic acid, with a grain or two of soda, in camphor or dill water. But in severe cases with much depression, and in many cases, as an alternative treatment, bismuth and creosote together will be found of eminent value.—*The Medical and Surgical Reporter.*

*Offensive Discharges from Cancer, etc.*—To counteract these, washing the parts with a weak solution of sulphurous acid; bathing them with terebene; and with a solution of chloral (dr.j to dr.iv), have been recommended.—*The Medical and Surgical Reporter.*

*Closing Cracks in Cast Iron Stoves.*—Good wood ashes are to be sifted through a fine sieve, to which is to be added the same quantity of clay finely pulverized, together with a little salt. The mixture is to be moistened with water enough to make a paste, and the crack of the stove filled with it. The cement does not peel off or break away, and assumes an extreme degree of hardness after being heated. The stove must be cool when the application is made. The same substance may be used in setting the plates of a stove, or in fitting stove pipes, serving to render all the joints perfectly tight.—*Scientific American.*

